# Does Cocaine Use Increase Medication Noncompliance in Bipolar Disorders? A United States Nationwide Inpatient Cross-Sectional Study

**DOI:** 10.7759/cureus.16696

**Published:** 2021-07-28

**Authors:** Gibson O Anugwom, Adeolu O Oladunjoye, Tajudeen O Basiru, Egbebalakhamen Osa, David Otuada, Victoria Olateju, Solomon Babalola, Olubunmi Oladunjoye, Maria Ruiza Yee, Eduardo D Espiridion

**Affiliations:** 1 Psychiatry and Behavioral Sciences, West Oaks Behavioral Hospital, Houston, USA; 2 Psychiatry and Behavioral Sciences, Houston Behavioral Healthcare Hospital, Houston, USA; 3 Psychiatry, Baylor College of Medicine, Houston, USA; 4 Medical Critical Care, Boston Children's Hospital, Boston, USA; 5 Developmental Behavioral Pediatrics, Dell Children's Medical Center, Austin, USA; 6 Psychiatry, Essen Health Care, New York, USA; 7 Psychiatry, Reading Hospital Tower Health, West Reading, USA; 8 Internal Medicine, Washington Adventist University, Takoma Park, USA; 9 Internal Medicine, Rockville Medical Care, Rockville, USA; 10 Psychiatry, University of Texas Health Science Center at Houston, Houston, USA; 11 Internal Medicine, Reading Hospital Tower Health, West Reading, USA; 12 Psychiatry, Drexel University College of Medicine, Philadelphia, USA; 13 Psychiatry, Philadelphia Collge of Osteopathic Medicine, Philadelphia, USA; 14 Psychiatry, West Virginia School of Osteopathic Medicine, Lewisburg, USA; 15 Psychiatry, West Virginia University School of Medicine, Martinsburg, USA; 16 Psychiatry, Philadelphia College of Osteopathic Medicine, Philadelphia, USA

**Keywords:** bipolar disorder, cocaine use, medication noncompliance, medication adherence, hospitalization

## Abstract

Introduction

Medication noncompliance among bipolar disorder (BD) is often linked with comorbid substance use disorders. This study aims to investigate cocaine use (CU) association with medication noncompliance in hospitalized BD patients.

Methods

Using data on 266,303 BD hospitalizations between 2010-2014 from the US Nationwide Inpatient Sample database, we obtained medication noncompliance rates stratified by demographics and cocaine use. Logistic regression was used to evaluate factors associated with medication noncompliance.

Results

Overall mean age, the prevalence of CU, and medication noncompliance were 41.58 (+0.11) years, 8.34%, and 16.08%, respectively. More than half of BD patients with comorbid CU were between 40-64 years (54.4%), while more male patients with BD were in the CU group (53.9%). With univariable logistic regression, CU (odds ratio [OR]: 1.77, 95% CI: 1.66-1.88) increased the odds of medication noncompliance among BD patients, and after adjusting for other variables there was sustained increased odds (adjusted odds ratio [aOR]: 1.40, 95% CI: 1.32-1.50).

Conclusion

This study showed that CU is associated with medication noncompliance among hospitalized BD patients. This highlights the importance of addressing CU among BD patients. Given the possible association of CU with medication noncompliance among BD patients, collaborative work between general adult psychiatry and addiction services is imperative in improving the management outcome of BD patients with comorbid CU.

## Introduction

Bipolar disorder (BD) is a serious mental health condition characterized by alternating episodes of mood swings, including manic and depressed episodes. Its exact cause is unknown. Bipolar disorder affects over 1% of the global population and 2.8 % of the US population. BD type I affects men and women equally, while BD type II affects more women than men [1, 2]. Considerable effort has been expended in the development of pharmacological and nonpharmacological management of BD, which has led to the use of medications such as lithium, antipsychotics, anticonvulsants, and newer treatment modalities like ketamine therapy and transcranial magnetic stimulation (TMS). These successes have been met with challenges, which in many ways, have limited the treatment outcomes. One of these challenges is medication noncompliance [3]. In comparison with other psychiatric pathologies, BD has one of the highest reported rates of medication noncompliance [4].

Factors associated with medication noncompliance in BD patients include adverse effects of medication, complex medication regimens, negative patient attitude to medications, poor insight, rapid cycling BD, comorbid substance misuse, and poor therapeutic alliance [5]. Medication noncompliance in BD patients carries a high risk of relapse due to the chronic and episodic nature of the illness [5]. 

Substance use disorder comorbidity is more common with BD than other psychiatric illnesses [6]. Patients with BD and comorbid substance use disorders have been found to experience an extensive array of psychopathological issues that are not commonly found in BD patients without co-diagnoses of substance use disorders [6]. Other studies have also identified medication noncompliance as a significant concern in BD patients with a current or previous history of substance use disorder [7]. For instance, a study by Montes et al. reported a significant association of medication nonadherence with substance use in BD patients [8]. In addition, Manwani et al. compared BD patients with and without comorbid substance use. They found a lower compliance rate in those with comorbid substance use [9]. However, to the best of our knowledge, no study has assessed the relationship of medication noncompliance with CU in BD patients using a large nationally representative database like the National Nationwide Inpatient Sample (NIS) database.

## Materials and methods

Study design and data sources

We conducted a retrospective study based on the NIS data administered by the Agency for Healthcare Research and Quality, a part of the Health Care Cost and Utilization Project (HCUP) [10]. The NIS is the largest epidemiological database involving inpatient data. We analyzed all adult admissions (18 years and above) from January 1, 2010, to December 31, 2014. International Classification of Diseases Ninth Revision Clinical Modification (ICD-9-CM) was derived from 25-30 diagnoses columns which were used to identify the study population. International Classification of Diseases Tenth Revision codes were not used because they were introduced in 2015. Since the database is de-identified and publicly available, ethical clearance or Institutional Review Board approval was unnecessary. The data used in this study can be accessed from the US Department of Health and Human Services website.

Study population and characterization of variables

We included 266,303 inpatient hospitalizations (ages 18 and above) from the NIS database with a primary ICD-9 diagnosis of bipolar disorders (296.XX) (Table1). We identified ICD-9 diagnosis codes for cocaine use (CU) and other substance use disorders (Table 1). We also identified patients based on medication noncompliance using ICD-9 code: V15.81. 

Patient demographics and other variables

Patient-level characteristics from the database included age (sub-divided into 18-24, 25-39, 40-64, and 65 + years), race (white, black, and others), primary insurance payer (government, private, self-pay, and others), zip code-based annual median household income (divided into four quartiles), and regions of the US (northeast, south, midwest/north-central, and west). Other variables extracted from the database include substance use (cocaine, cannabis, stimulants, alcohol, hallucinogens) and medication compliance.

Statistical analysis

The prevalence of medication non-compliance and CU were identified among BD patients. We used bivariate analysis to compare demographics and substance use disorders in patients with BD by medication noncompliance. Multivariable logistic regression analyses adjusted for demographics were used to evaluate the factors related to medication noncompliance in BD patients. STATA version 15.0 (College Station, TX) was used for all statistical analyses. We used a p-value of <0.05 and a 95% confidence interval (CI).

**Table 1:** ICD-9 diagnostic codes ICD-9: International Classification of Diseases Nine

Diagnosis	ICD-9 diagnostic code
Alcohol	303.00, 303.01, 303.02, 303.03, 303.90, 303.91, 303.92, 303.93, 305.00, 305.01, 305.02, 305.03, 980.0, 980.1, 980.2, 980.3, 980.8, 980.9
Sedative-hypnotic	304.10, 304.11, 304.13, 305.40, 305.41, 305.42, 305.43, 967.0, 967.1, 967.2, 967.3, 967.4, 967.5, 967.6, 967.8, 967.9, 969.4, E851, E852.0, E852.1, E852.2, E852.3, E852.4, E852.5, E852.8, E852.9, E853.0, E853.1, E853.2, E853.8, E853.9, E937.0, E937.1, E937.2, E937.3, E937.4, E937.4, E937.5, E937.6, E937.7, E937.8, E937.9
Cocaine	304.20, 304.22, 304.23, 305.60, 305.61, 305.62, 305.63, 970.81, 970.89
Stimulant	304.00, 304.41, 304.42, 304.43, 305.70, 305.71, 305.72, 305.73, 969.6, 970.0, 970.1, 970.9, E854.2, E854.3, E854.9
Hallucinogen	304.50, 304.51, 304.52, 304.53, 305.30, 305.31, 305.32, 305.53, E854.1, E855.5, E855.6, E855.8, E855.9
Cannabis	304.30, 304.31, 304.32, 305.20, 305.21, 305.22
Bipolar disorder	296.40, 296.41, 296.42, 296.43, 296.44, 296.50, 296.51, 296.52, 296.53, 296.54, 296.60, 296.61, 296.62, 296.63, 296.64, 296.7, 296.80, 296.89
Medication noncompliance	V15.81

## Results

A total of 266,303 hospitalizations with BD were analyzed from 2010 to 2014, with an overall mean patient age of 41.58 ± 0.11 years. In this population, the prevalence of CU was 8.34%, while that of medication noncompliance was 16.08%. They were subclassed based on CU with a mean age of 40.33 ± 0.07 years in the CU group (Table 2). More than half of the BD patients in the CU group were between the ages of 40-64 years (54.4%).

**Table 2:** Baseline and Characteristics of Bipolar Disorder Inpatients by Cocaine Use n: sample number; SE: Standard error; %: percentage; CU: Cocaine Use

Name	Overall (n= 266,303)	Non-CU (n=244,080)	CU (n=22,223)	P Value
Mean Age (±SE)	41.58 ± 0.11	41.70 ± 0.03	40.33 ± 0.07	<0.001
Age, years				
18-24	14.5	15.1	8.2	
25-39	32.8	32.4	36.8	
40-64	45.2	44.4	54.4	
≥ 65	7.5	8.1	0.6	<0.001
Sex, %				
Female	56.6	57.6	46.1	
Male	43.4	42.4	53.9	<0.001
Race, %				
White	72.9	74.5	55.8	
Black	14.8	13.3	31.7	
Others	12.3	12.2	12.5	<0.001
Substance use disorder				
Cannabis Use	15.1	13.4	33.0	<0.001
Stimulant	3.2	2.9	6.7	<0.001
Hallucinogen	0.2	0.2	1.0	<0.001
Alcohol Use	22.3	20.0	46.7	<0.001
Sedative	4.0	3.8	7.1	<0.001
Income, %				
First quartile	32.6	31.7	42.8	
Second quartile	26.6	26.8	24.1	
Third quartile	22.4	22.8	18.8	
Fourth quartile	18.3	18.7	14.3	<0.001
Insurance, %				
Government	60.3	59.6	67.2	
Private	25.1	26.0	15.6	
Self- Pay	8.7	8.5	11.0	
Others	5.9	5.9	6.2	<0.001
Region, %				
North East	22.3	21.9	26.2	
Mid-West/North Central	27.7	27.8	27.6	
South	36.3	36.2	37.8	
West	13.7	14.1	8.4	<0.001
Hospital Teaching Status, %				
Rural	10.8	11.2	6.3	
Urban non-teaching	40.8	40.9	39.1	
Urban teaching	48.4	47.9	54.6	<0.001

Table 2 describes the demographic and clinical characteristics of hospitalizations among BD patients by CU. More females were in the overall population and the non-CU group (56.6% and 57.6%, respectively), while more males were in the CU group (53.9%). The population of patient hospitalizations was predominantly white across the board, with an overall proportion of 72.9%. Comorbid substance use among CU patients varied across the population, with alcohol use being the predominant substance (46.7%) followed by cannabis use (33.0%) and sedatives (7.1%). Insurance coverage varied, with government insurance (Medicare and Medicaid) being the predominant coverage (60.3%), followed by private insurance (26.6%) and other types of insurance. About 36.3% of the patients were from the southern part of the country, and 32.6% were from areas with a national average income below the 25th percentile. 

Factors associated with medication noncompliance in hospitalized bipolar disorder patients

Table 3 describes the demographic and clinical characteristics of hospitalizations among BD patients by medication noncompliance. There was a significant difference between the characteristics of the medication noncompliance group and those that were medication compliant in terms of age, gender, race, comorbid substance use, income, insurance type hospital region, and hospital teaching status (p<0.001).

**Table 3:** Baseline and clinical characteristics of Bipolar Disorders inpatients by Medication Compliance n: sample number; SE: Standard error; %: percentage

Name	Overall (n= 266,303)	Non-compliance (n= 42,823)	Compliance (n= 223,480)	P Value
Mean Age (±SE)	41.58 ± 0.11	41.34 ± 0.07	41.63 ± 0.03	<0.001
Age, years				
18-24	14.6	14.0	14.6	
25-39	32.8	32.9	32.8	
40-64	45.3	47.0	44.9	
≥ 65	7.4	6.1	7.7	<0.001
Sex				
Female	56.6	50.8	57.8	
Male	43.4	49.2	42.2	<0.001
Race, %				
White	72.9	60.7	75.3	
Black	14.8	25.2	12.8	
Others	12.3	14.1	11.9	<0.001
Substance use disorder				
Cannabis Use	15.1	20.1	14.0	<0.001
Cocaine Use	8.4	12.6	7.6	<0.001
Stimulant	3.2	3.5	3.2	0.032
Hallucinogen	0.2	0.3	0.2	0.008
Alcohol Use	22.2	25.9	21.6	<0.001
Sedative	4.0	3.5	4.1	0.003
Income, %				
First quartile	32.6	35.2	32.2	
Second quartile	26.6	24.5	27.0	
Third quartile	22.4	21.5	22.6	
Fourth quartile	18.3	18.8	18.2	<0.001
Insurance, %				
Government	60.3	67.0	59.0	
Private	25.1	18.9	26.3	
Self- Pay	8.7	8.7	8.7	
Others	45.9	5.4	6.0	<0.001
Region, %				
North East	22.3	27.8	21.2	
Mid-West/North Central	27.7	26.4	28.0	
South	36.3	33.0	37.0	
West	13.7	12.8	13.8	<0.001
Hospital Teaching Status, %				
Rural	10.8	7.7	11.4	
Urban non-teaching	40.8	39.3	41.0	
Urban teaching	48.4	53.0	47.6	<0.001

With univariate analysis, the following factors were associated with medication noncompliance: ages 25-39 years compared to ages 18-24 years (OR: 1.09, 95% CI: 1.04-1.14, p<0.001), Blacks compared to the white race (OR: 2.44, 95% CI: 2.18-2.72, p<0.001), comorbid cocaine use compared to nonuse (OR: 1.77, 95%CI: 1.66-1.88, p <0.001) (Table 4). Females were at reduced odds of medication noncompliance compared to males (OR: 0.75, 95% CI: 0.73-0.78, p<0.001). After adjusting for other covariates using multivariate analysis, there remained a statistically significant association of medication noncompliance in BD hospitalizations and comorbid cocaine use (OR: 1.41, 95% CI: 1.32-1.50; p<0.001) (Table 4). Blacks compared to whites (OR: 2.22, 95% CI: 1.99-2.47), ages 40-64 years compared to 18-24 years (OR: 1.05, 95% CI: 1.00-1.10) and urban centers compared to rural centers (urban nonteaching (OR: 1.35, 95%CI: 1.15-1.59); urban teaching (OR: 1.43, 95%CI: 1.23-1.65; p<0.001) also remained statistically significant.

**Table 4:** Factors associated with medication noncompliance in hospitalized patients with bipolar disorders SE: Standard error; %: percentage; ref: reference

Name	Univariate analysis (Crude OR)	P-Value	Multivariate analysis (Adjusted OR)	P-Value
Mean Age (±SE)	1.00 (1.00-1.00)	0.022		
Age, years				
18-24	Ref			
25-39	1.05 (1.00-1.09)	0.029	1.01 (0.97-1.06)	0.567
40-64	1.09 (1.04-1.14)	<0.001	1.05 (1.01-1.10)	0.039
≥ 65	0.83 (0.78-0.89)	<0.001	0.86 (0.80-0.93)	<0.001
Sex				
Male	Ref			
Female	0.75 (0.73-0.78)	<0.001	0.78 (0.76-0.81)	<0.001
Race, %				
White	Ref			
Black	2.44 (2.18-2.72)	<0.001	2.22 (1.99-2.47)	<0.001
Others	1.47 (1.36-1.59)	<0.001	1.37 (1.27-1.48)	<0.001
Substance use disorder				
Cocaine Use	1.77 (1.66-1.88)	<0.001	1.40 (1.32-1.50)	<0.001
Income, %				
First quartile	Ref			
Second quartile	0.83 (0.78-0.88)	<0.001	0.96 (0.91-1.02)	0.208
Third quartile	0.87 (0.80-0.93)	<0.001	0.99 (0.92-1.07)	0.884
Fourth quartile	0.94 (0.85-1.04)	0.251	1.11 (1.00-1.23)	0.046
Insurance, %				
Government	Ref			
Private	0.63 (0.59-0.67)	<0.001	0.67 (0.64-0.71)	<0.001
Self- Pay	0.88 (0.80-0.97)	0.009	0.89 (0.81-0.98)	0.018
Others	0.79 (0.72-0.86)	<0.001	0.83 (0.75-0.91)	<0.001
Region, %				
North East	Ref			
Mid-West/North Central	0.72 (0.60-0.86)	<0.001	0.77 (0.64-0.91)	0.002
South	0.68 (0.59-0.78)	<0.001	0.72 (0.62-0.82)	<0.001
West	0.70 (0.59-0.85)	<0.001	0.77 (0.64-0.93)	0.008
Hospital Teaching Status, %				
Rural	Ref			
Urban non-teaching	1.42 (1.21-1.67)	<0.001	1.35 (1.15-1.59)	<0.001
Urban teaching	1.65 (1.42-1.88)	<0.001	1.43 (1.23-1.65)	<0.001

## Discussion

The findings in this study show that the medication noncompliance rate among hospitalized BD patients was 16%. While few studies have examined medication compliance among hospitalized BD patients, medication noncompliance rates vary considerably across the literature [11], with most studies reporting over 40% nonadherence rates among BD patients [12]. A study that asked European psychiatrists about medication non-compliance among BD patients found an estimated rate of 57% [13]. An article published in the World Journal of Psychiatry supports the high variability in the reported adherence rates because these rates depend on the settings where the studies were conducted. Some specialized settings, such as hospitalized patients, as in our study, may report very low rates of medication noncompliance [9, 14], while others may report higher rates [3].

In the bivariate analysis (Table 3), BD patients that used cocaine and other substances were significantly more likely to be non-adherent with medication (p<0.05). In the logistic regression analysis, cocaine use was significantly associated with an increased risk of medication noncompliance after adjusting for other factors (aOR: 1.40, 95% CI: 1.32-1.50, p <0.001). Few studies have examined the association of CU with medication noncompliance among BD patients. However, the relationship between substance use and BD is well established in the literature [5]. Many studies have found substance abuse (including cocaine) to be significantly associated with a higher risk of medication noncompliance in BD patients [15, 16]. The current study is unique in that it examines the association of CU with medication noncompliance in hospitalized BD patients using nationally representative data.

We found that the use of other substances (cannabis, stimulants, hallucinogens, alcohol, and sedatives) by BD patients with comorbid CU was about twice the rate of use of similar substances in BD patients without comorbid CU. This is an expected finding because people that use one substance are at higher risk for polysubstance use, and other studies have shown the association of polysubstance abuse with many psychiatric illnesses, including BD [17]. The lifetime prevalence of comorbidity of any BD with any substance use disorder is said to be over 47%, and up to 60% for bipolar I disorder [18]. The relationship between BD and substance use is suggested by multiple research to be bidirectional [19]. 

In our study, 31.7% of non-CU BD patients were in the lower-income group (first quartile), compared to over 40% of BD patients with comorbid CU. In addition, while 67.2% of the CU group had government insurance, the proportion of non-cocaine users with government insurance was less than 60%. These findings suggest that BD patients who use cocaine are likely to be financially constrained than those who do not use cocaine. This may also explain why more patients in this group may prefer to use government insurance as it is more affordable than private insurance. There is also a possibility of greater use of government resources among those that use cocaine than those that do not use cocaine. Although there are limited studies on the relationship between personal finances and substance use in BD patients, these findings seem to agree with some studies that suggest an adverse relationship and overall increased consumption of public resources among substance use patients [20, 21]. 

We found older age (age 65 years and older) to be significantly associated with a reduced risk of medication noncompliance compared to other age groups after controlling for other factors (95% CI= 0.78-0.89, p-value <0.001). This is similar to findings from studies that examined factors associated with medication noncompliance. For instance, Sajatovic et al. and Baldessarini et al. reported increased odds of medication noncompliance in younger age groups compared to older age groups [22, 23]. It is noteworthy that our study did not show a significant association between younger age groups and medication noncompliance among BD patients. Female gender was found to be associated with a lower risk of noncompliance in this study compared to male gender after controlling for other factors using multivariate analysis OR (95% CI) 0.78 (0.76-0.81), p-value <0.001. Many published studies did not find a statistically significant association between gender and medication noncompliance among BD patients [24, 25]. Of note, in the current study CU patients were also more likely to be male. Sajatovic et al. found that men with high masculinity index were four times more likely to be noncompliant with medication than female gender or males without high masculinity index [25].

From our study, race played a significant factor in medication noncompliance. While only 14.8% of patients in our study were Black, the proportion of Black patients among those not compliant was over 25%. In the multivariate analysis, we found that being Black and being from races other than white were associated with increased odds of medication noncompliance OR(95%CI) 2.44 (2.18-2.72), p<0.001 and 1.47 (1.36-1.59) respectively) which supports findings from previous studies [26, 27]. Sajatovic et al. that evaluated factors that influence medication nonadherence among a highly nonadherent group found that minorities such as Blacks were disproportionately represented [28].

Several poor outcomes have been linked with medication noncompliance in BD patients, including reduced treatment effectiveness, re-hospitalization, poor quality of life, relapse of symptoms, increased comorbid medical conditions, wastage of health care resources, increased suicide rates, and decreased likelihood of illness remission [29]. Some of the factors associated with medication noncompliance in these patients include adverse effects of medication, complex medication regimens, negative patient attitude to medications, poor insight, rapid cycling BD, comorbid substance misuse, and poor therapeutic alliance [5]. Among these factors, substance use disorder is estimated to be hugely responsible for most of the adverse outcomes in these patients [30]. 

A strength of this study is the use of large nationally representative data, which increases the power of the study and allows the generalization of findings to other patients in similar settings. However, one of the limitations in this study includes the fact that data of hospitalized patients were used, thereby limiting generalization of findings to the general population. Another limitation is the possibility of over- or underestimation of rates in using the NIS data because the data is derived from ICD codes that are vulnerable to administrative coding errors.

## Conclusions

Medication noncompliance in BD treatment is associated with multiple detrimental effects during BD management. The report that BD patients who have a comorbid CU are more likely to be medication noncompliance highlights the importance of addressing substance use and BD. A better understanding of strategies necessary to address these concerns might be a step in the right direction in caring for BD patients.

Given the possible association of CU and medication noncompliance among BD patients, collaborative work between general adult psychiatry and addiction services is imperative in improving the management outcome of BD patients with comorbid CU. 
